# A time-series analysis of morbidity and mortality of viral hepatitis in Venezuela, 1990–2016

**DOI:** 10.1186/s12879-023-08338-1

**Published:** 2023-05-27

**Authors:** Alejandro Rísquez, Luis Echezuría, Fhabián S. Carrión-Nessi, David A. Forero-Peña

**Affiliations:** 1 “Luis Razetti” School of Medicine, Central University of Venezuela, Caracas, Venezuela; 2 Biomedical Research and Therapeutic Vaccines Institute, Ciudad Bolivar, Venezuela; 3 Infectious Diseases Department, University Hospital of Caracas, Caracas, Venezuela

**Keywords:** Epidemiology, Viral hepatitis, Morbidity, Mortality, Venezuela

## Abstract

**Background:**

Viral hepatitis (VH) is a leading contributor to morbidity and mortality worldwide, constituting a public health problem associated with the level of human development. In recent years, Venezuela has experienced a political, social, and economic crisis and has been impacted by natural disasters that have led to the deterioration of sanitary and health infrastructures modifying the determinants of VH. Despite epidemiological studies conducted in specific regions of the country or populations, the national epidemiological behaviour of VH remains unclear.

**Methods:**

This is a time series study involving records of morbidity and mortality by VH in Venezuela reported during the period from 1990 to 2016. The Venezuelan population was taken as the denominator of the morbidity and mortality rates, according to the Venezuelan National Institute of Statistics and the 2016 population projections from the latest census published on the website of the responsible Venezuelan agency.

**Results:**

During the study period, 630,502 cases and 4,679 deaths from VH in Venezuela were analysed. Most of the cases (*n* = 457,278; 72.6%) were classified as unspecific VH (UVH). The deaths were mainly attributed to VHB (*n* = 1,532; 32.7%), UVH (*n* = 1,287; 27.5%), and sequelae of VH (*n* = 977; 20.8%). The mean rates of cases and deaths from VH in the country were 95 ± 40.4 cases per 100,000 inhabitants and 0.7 ± 0.1 deaths per 100,000 inhabitants, respectively, showing a large dispersion that is evident from the calculation of the coefficients of variation. There was document a strong correlation between UVH and VHA cases (0.78, *p* < 0.01) morbidity rates. VHB mortality rate was very strongly correlated with sequelae of VH (–0.9, *p* < 0.01).

**Conclusions:**

VH is a major burden of morbidity and mortality in Venezuela with an endemic-epidemic trend and an intermediate prevalence for VHA, VHB, and VHC. Epidemiological information is not published in a timely manner and diagnostic tests are insufficient in primary health services. There is an urgent need to resume epidemiological surveillance of VH and to optimise the classification system for a better understanding of UVH cases and deaths due to sequelae of VHB and VHC.

## Background

Viral hepatitis (VH) is a leading contributor to morbidity and mortality worldwide [1], constituting a public health problem associated with the level of human development [2]. The primary aetiology of VH is infection by a hepatotropic virus, including hepatitis A (HAV), B (HBV), C (HCV), D (HDV), and E (HEV) viruses [3]. Annually, there are an estimated 100 million HAV infections worldwide that are responsible for between 15,000 and 30,000 deaths per year [4]. In 2019, 296 million people were living with chronic HBV infection and 58 million people with chronic HCV infection worldwide. Furthermore, VH caused 1.1 million deaths in 2019, mainly from chronic complications such as cirrhosis and primary hepatocarcinoma, of which 96% were caused by HBV and HCV [5].

In 2016, the World Health Organisation (WHO) adopted the Global Health Sector Strategy on Viral Hepatitis [6], which aims to reduce new hepatotropic virus infections and their associated mortality by 90% and 65%, respectively, by 2030 through HBV vaccination, safe injection, damage reduction, and diagnosis and treatment of HBV and HCV. However, the main obstacle to achieving the elimination of these VH is the lack of funding for the creation or support of control programmes, particularly in low- and middle-income countries, such as some Latin American countries. The elimination of hepatitis viruses requires strong financial and political commitment, the support of civil society, and the support of pharmaceutical companies [7].

In recent years, Venezuela has experienced a political, social, and economic crisis and has been impacted by natural disasters that have led to the deterioration of sanitary and health infrastructures and long-term shortages of essential medicines and medical supplies, with only 30% of basic medicines to treat infectious diseases available in public hospitals [8–10]. Moreover, the collapse of epidemiological surveillance systems and the weakening of immunisation programmes have created the perfect scenario for the resurgence of vector-borne and vaccine-preventable diseases [11]. However, analysis of the impact of this complex crisis has been limited due to the absence of official epidemiological bulletins and mortality yearbooks in the country since 2016.

Between 1990 and 1999, Venezuela reported an HAV and HBV prevalence of 24.2 cases and 3.6 cases per 100,000 inhabitants, respectively, with a special attack rate in the paediatric age group [12]. In Venezuelan high-risk populations such as sex workers, Camejo *et al*. [13] in 1999 documented a seroprevalence of 3.8% for both HBsAg and anti-HBc, while that of anti-HCV was 0.5%. More recently, in 2017, a study of the Warao indigenous communities of the Orinoco Delta, Venezuela documented a seroprevalence of HBsAg of 1.8% and anti-HBc of 13% [14]. In 2022, a study of Venezuelan and Haitian migrants and refugees in Brazil reported an anti-HAV immunoglobulin G prevalence of 94.9% in Haitians and 75.6% in Venezuelans aged 19 years and over [15].

Despite epidemiological studies conducted in specific regions of the country or populations, the national epidemiological behaviour of VH remains unclear. We assess here the trends of VH and its types in Venezuela between 1990 and 2016 through a time-series study of cases and deaths.

## Methods

### Study design and population

This is a time series study involving records of morbidity and mortality by VH in Venezuela reported during the period from 1990 to 2016. The Venezuelan population was taken as the denominator of the morbidity and mortality rates, according to the Venezuelan National Institute of Statistics and the 2016 population projections from the latest census published on the website of the responsible Venezuelan agency [16].

### Study site

The setting for this study was Venezuela with its 25 states. The country has a total geographical area of 916,445 km^2^, located in the northern part of South America, with a 2016 projected population of approximately 31,028,637 inhabitants [16]. Venezuela is characterised by significant regional inequalities that have been accentuated by the recent political, social, and economic crisis [17] and have precipitated the emergence and re-emergence of vector-borne and immuno-preventable diseases [11, 18].

### Venezuela’s epidemiological surveillance system

The structure, design, and operation of the Venezuelan health system is an official agency whose objective is to monitor morbidity and mortality occurred in the national territory through the “Ministerio del Poder Popular para la Salud” (Ministry of Health).

In relation to morbidity, there are the Mandatory Notifiable Diseases (infectious and non-infectious diseases) that are classified according to their periodicity of reporting in immediate, daily, or weekly notification (in the case of hepatitis, the report is weekly) [19]. Notification, denunciation, and reporting are routinely and mandatorily performed by law and depend on the physicians of the medical care units, discriminated into urban outpatient clinics, rural outpatient clinics, and all types of hospitals including specialised ones. The information obtained by these units must be systematically and thoroughly collected by local or regional epidemiologists and subsequently used to generate the Venezuelan Epidemiological Bulletins.

Furthermore, death statistics come from the death certificates registered continuously in the municipalities, civil registry units, prefectures, and civil headquarters throughout a calendar year. The country’s information sources report each month the deaths registered in the previous month, based on the death certificates, which are filled out the first five days of each month by officials of the National Institute of Statistics and subsequently published in the mortality yearbooks. The quality of death registration data obtained from the Venezuelan epidemiological surveillance system has been previously rated as high [20].

### Variables and data sources

In this study, data were extracted from Epidemiological Bulletins (not published since 2016) and official yearbooks published from 1990 to 1995 in printed physical version and from 1996 to 2016 in digital version from the portal page of the responsible Venezuelan agency [21]. No population was excluded. All ages and all sexes were included. The specific diagnoses of VH according to duration, acute or chronic, or according to viral type (A, B, C, D or E) are clinical, epidemiological, or laboratory-based. Unspecific viral hepatitis (UVH) are all those VH where the diagnosis does not specify the viral type either by clinical diagnosis, presumptive epidemiological diagnosis, or confirmation by paraclinical tests. This includes VH without further specification, as listed in the WHO’s International Statistical Classification of Diseases and Related Health Problems, Tenth Revision (ICD-10) [22].

The Venezuelan mortality yearbooks use the coding of the ICD-10 since 1996: VH (B15–B19) and sequelae of VH (B94.2), referred to for the purpose of this paper as total viral hepatitis (TVH) (B15: acute hepatitis type A; B16: acute hepatitis type B; B17: other acute viral hepatitis; B18: chronic viral hepatitis; B19: unspecific viral hepatitis) [22]. The coder from the mortality statistics department analysed the certificates according to the causes of death recorded (direct cause, antecedent causes, and basic cause) and the approximate time interval between the onset of the disease and death. Mortality due to HCV infection and sequelae of VH were incorporated into the yearbooks in 1996, while cases of VHC were incorporated into the epidemiological bulletins from 2005 onwards. Moreover, in 2004–2005 there was a change in coding in the statistical section of the Ministry of Health (where ICD-10 coding is assigned, in terms of the time of VH and sequelae of VH according to the basic cause of VH).

### Statistical analysis

Statistical analysis was performed using Statistical Package for the Social Sciences version 21 (International Business Machines Corporation, Armonk, NY, USA) and figures was generated using Microsoft® Excel® version 2019 (Microsoft, Redmond, WA, USA). For the calculation of rates, the nominator was the specific statistics from Epidemiological Bulletins and official yearbooks in absolute numbers per year, and the denominator was the specific population according to official data from the Venezuelan National Institute of Statistics, multiplied by a constant of 100,000 inhabitants.

The variables were analysed with absolute and relative values, calculation of year-specific rates, averages of lapses, and indices. For the calculation of average rates, triennia were used from the first three chronological years of the series in ascending order. The percentage rate was calculated based on 100% of the first data in the time series in consecutive order. The annual case fatality rate was calculated as a division between annual mortality and morbidity and is expressed as a percentage.

For the study of variable correlations, Pearson’s correlation coefficient was used with a confidence level of 95% and 99%, α error = 0.05 and 0.01, respectively. R value > 0.8 was considered very strong; 0.6 to 0.79, strong; 0.4 to 0.59, moderate; 0.2 to 0.39, weak; and < 0.2, absent. The polynomial trend line for VHB and sequelae of VH mortality was plotted, and the formula equation and coefficient of determination (R^2^) were calculated.

## Results

During the study period, 630,502 cases and 4,679 deaths from VH in Venezuela were analysed. A total of 457,278 cases (72.5%) were classified as UVH, 140,511 (22.3%) as VHA, 29,785 (4.7%) as VHB, and 2,918 (0.5%) as VHC. Moreover, a total of 1,532 deaths (32.7%) were attributed to VHB, 1,287 (27.5%) to UVH, 977 (20.9%) to sequelae of VH, 643 (13.8%) to VHC, and 240 (5.1%) to VHA. The fatality rate for VH was 0.9% (SD —standard deviation— 0.4), with significant differences in all types of VH: the highest was 11.6% (SD 5) for VHC, followed by 5.4% (SD 2.6) for VHB, 0.3% (SD 0.2) for UVH, and 0.2% (SD 0.1) for VHA.

The mean rates of cases and deaths from VH in the country were 95 ± 40.4 cases per 100,000 inhabitants and 0.7 ± 0.1 deaths per 100,000 inhabitants, respectively, showing a large dispersion that is evident from the calculation of the coefficients of variation, which are very wide for all morbidity and mortality averages for TVH. This variation is between 32% and 46% for morbidity and rates ranging from a maximum of 181 cases per 100,000 inhabitants in 2005 to a minimum of 39 cases per 100,000 inhabitants in 2014 (Table 1).

**Table 1:** Annual morbidity and mortality rates (× 100,000 inhabitants) by type of VH in Venezuela

**Type of VH**	**Years analysed**	**Mean**	**Standard deviation**	**Minimal**	**Maximum**	**Coefficient of variation**
*Morbidity*						
VHA	27	20.9	9.5	9.4	56.4	45.6
VHB	27	4.4	1.4	1.8	6.9	32.5
VHC	12	0.9	0.3	0.3	1.2	32.7
UVH	27	69.4	32.3	25.9	120.6	46.5
TVH cases	27	94.9	40.4	38.8	181.3	42.6
*Mortality*						
VHA	27	0	0.01	0.01	0.1	46.6
VHB	27	0.2	0.1	0.1	0.5	52.6
VHC	21	0.1	0.1	0.04	0.2	49.6
UVH	27	0.2	0.1	0.1	0.5	62.2
Sequelae of VH	21	0.2	0.1	0	0.4	89.1
TVH deaths	27	0.7	0.1	0.5	0.9	14.5

*VH* Viral hepatitis, *VHA* Viral hepatitis A, *VHB* Viral hepatitis B, *VHC* Viral hepatitis C, *UVH* Unspecific viral hepatitis, *TVH* Total viral hepatitis

**Fig. 1:**
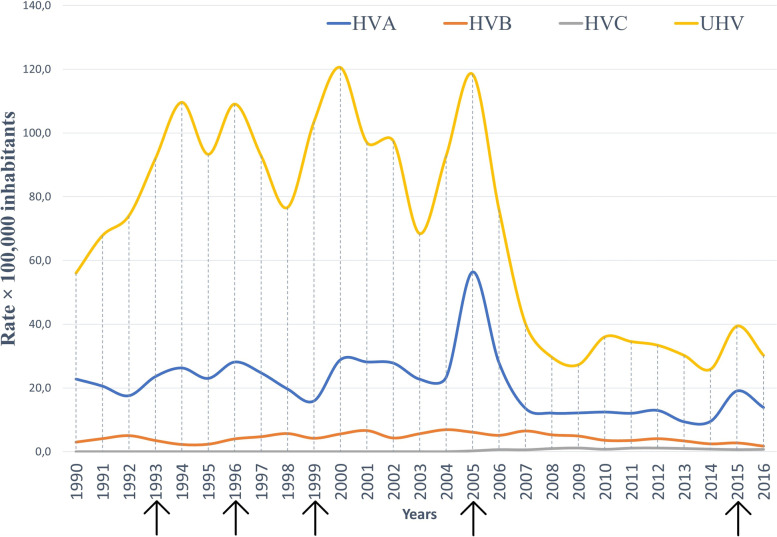
Annual morbidity rates (× 100,000 inhabitants) by type of VH in Venezuela, 1990–2016. The arrows (black) show the catastrophic events due to floods and landslides in Venezuela (1993: Storm Bret, which affected 11,219 people in the Libertador municipality [Distrito Capital] and caused 84 deaths; 1999: Tragedy of Vargas, which affected 267,462 people in the Vargas municipality [Vargas state] and caused more than 758 deaths; 2005: Overflow of the Mocotíes river, which affected 16,000 people in the Antonio Pinto Salinas municipality [Mérida state] and caused 48 deaths). VHA: viral hepatitis A; VHB: viral hepatitis B; VHC: viral hepatitis C; UVH: unspecific viral hepatitis

**Fig. 2:**
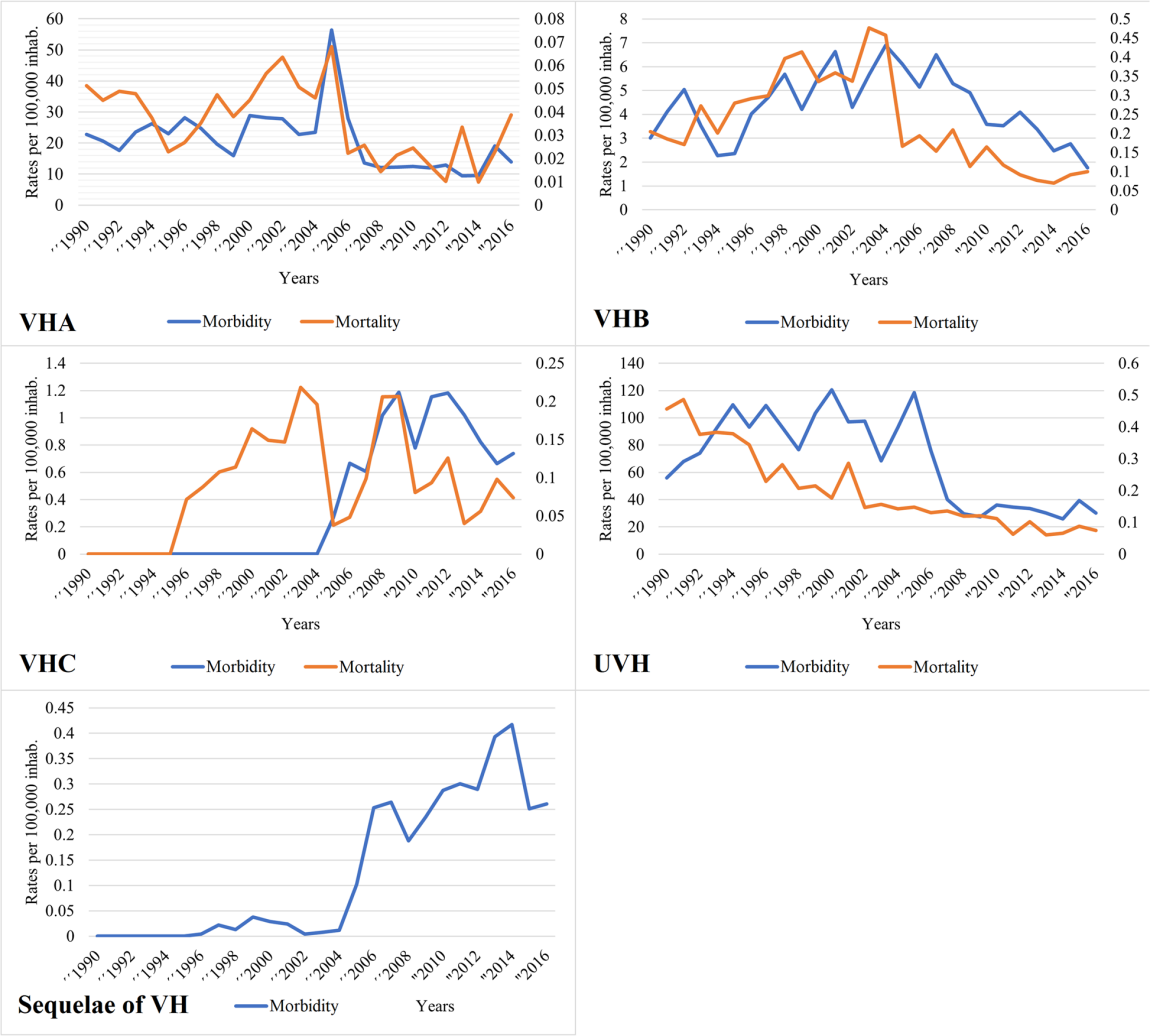
Annual morbidity and mortality rates (× 100,000 inhabitants) by type of VH in Venezuela, 1990–2016. VHA: viral hepatitis A; VHB: viral hepatitis B; VHC: viral hepatitis C; UVH: unspecific viral hepatitis

Figure 1 shows the behaviour of VH morbidity during the study period, evidencing peaks of epidemic wave to UVH in 1994, 1996, 2000, 2005, and 2015, with a subsequent continuous decrease in the last subsequent years (span from 2006 to 2014) for the VH. The epidemiologic behaviour of VHA was very similar to that of UVH. However, a more accentuated decrease is observed for UVH than for VHA. While VHB and VHC had a less variable behaviour throughout the study period. Figure 2 shows the morbidity and mortality rates by type of VH during the study period, showing that the curves have different behaviours and trends. The mortality rates for VHB and UVH decreased significantly, while the mortality rate for sequelae of VH increased significantly since 2014, same year in which VHB declines dramatically. VHA remained relatively stable over time, while VHC was more oscillating with a decreasing trend since 2009.

There was very strong correlation between VHA and TVH (0.87, *p* < 0.01) morbidity rates, and strong correlation between VHA and VHC (–0.7, *p* < 0.05) and UVH (0.78, *p* < 0.01) morbidity rates. Likewise, there was strong correlation between VHC and UVH (–0.7, *p* < 0.05) and TVH (–0.7, *p* < 0.05) morbidity rates, and there was very strong correlation between UVH and TVH (0.99, *p* < 0.01) morbidity rates. Furthermore, there was strong correlation between VHA and sequelae of VH (–0.74, *p* < 0.01) mortality rates, and moderate correlation between VHA and VHB (0.55, *p* < 0.01) and UVH (0.46, *p* < 0.05) mortality rates. VHB mortality rate was very strongly correlated with sequelae of VH (–0.9, *p* < 0.01) and TVH (0.82, *p* < 0.01) mortality rates, and moderately correlated with VHC (0.5, *p* < 0.05) mortality rate. VHC mortality rate was strongly correlated with TVH (0.71, *p* < 0.01) mortality rate, while UVH mortality rate was very strongly correlated with sequelae of VH (–0.81, *p* < 0.01) mortality rate. There was strong correlation between sequelae of VH and TVH (–0.63, *p* < 0.01) mortality rates.

**Fig. 3:**
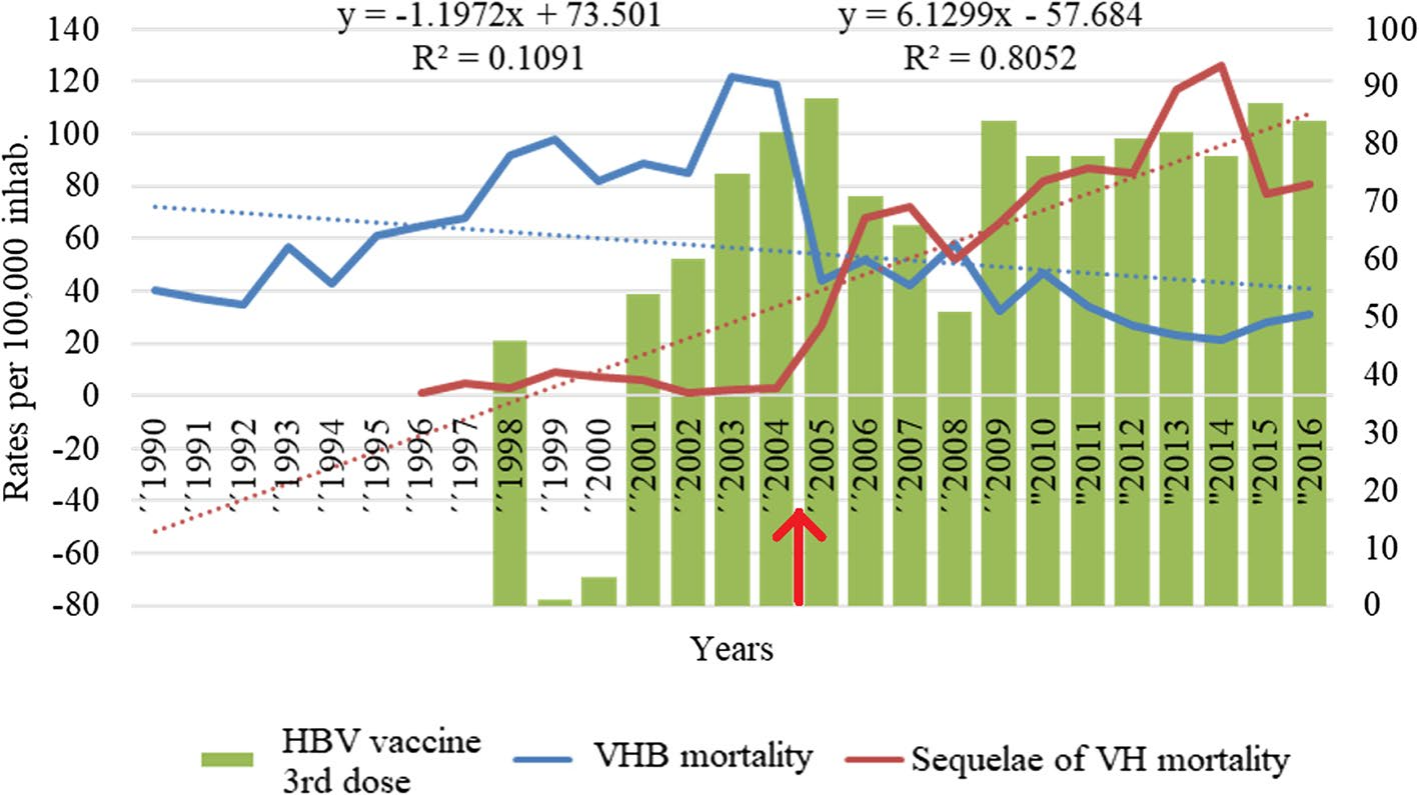
Annual mortality rates (× 100,000 inhabitants) by VHB and sequelae of VH and HBV vaccine 3^rd^ dose coverage (%) in children under one year of age, in Venezuela, 1990–2016 [23]. The arrow (red) shows when a coding change occurred in the statistical section of the Ministry of Health. HBV: hepatitis B virus; VHB: viral hepatitis B

To analyse the correlation between mortality due to VHB and sequelae of VH, a polynomial trend line was plotted, and the formula equation and coefficient of determination (R^2^) were calculated. A decrease in deaths due to VHB and an increase in deaths due to sequelae of VH were observed from 2005 onwards. Furthermore, hepatitis B vaccination coverage rates in children under one year of age were plotted, showing a progressive increase from 2001 to an all-time high of 88% in 2005 [23] (Fig. 3).

When reviewing mortality yearbooks for the sequelae of VH, the majority of deaths from malignant tumours of the liver and intrahepatic bile ducts (C22) and from non-alcoholic liver fibrosis and cirrhosis (K74) occurred in adults older than 44 years (90%), suggesting that these deaths are a consequence of chronic VH, according to ICD-10 (Table 2).

**Table 2:** Mortality from sequelae of VH by age group in Venezuela, 2014–2016

**Years**	**Sequelae of VH**	**<10 years old**	**10–19 years old**	**20–34 years old**	**35–59 years old**	**≥60 years old**
2014	126	0	1	2	56	67
2015	77	1	0	3	39	34
2016	81	0	1	3	30	47
Total (%)	284 (100)	1 (0.4)	2 (0.7)	8 (2.8)	125 (44)	148 (52.1)

*VH* Viral hepatitis

## Discussion

The endemic curve of average annual morbidity and mortality rates for VH shows an overall endemic-epidemic behaviour with epidemic waves of morbidity preceding mortality in the period from 1999 to 2016. Subsequently, both rates seem to fall progressively, being more important in mortality rates. Morbidity explains, in acute infectious pathologies, mortality trends, as occurs in the endemic VH curve, a proportion of which is acute, which may impact on sequencing [24]. The progressive general decrease in morbidity and mortality from VH (except for sequelae of VH) in the last years analysed in this time series could be partly explained not only by the above-described change in reporting, but also by the gradual deterioration of the epidemiological surveillance system as a consequence of the humanitarian and health system crisis in Venezuela, with epidemiological bulletins and mortality yearbooks even disappearing completely from 2016 onwards.

### VHA, catastrophic events due to floods and landslides in Venezuela, and low VHA vaccination rates

VHA infection is closely associated with unsanitary food and water consumption, poor sanitation, and poor personal hygiene [4]. In this study, epidemic behaviour of VHA and UVH match with the large troughs and landslides caused by torrential rains that occurred during those years in several regions and localities of the country. The activity of tropical storm Bret in 1993 caused floods and material losses through Venezuelan territory, including Monagas, Sucre, Nueva Esparta, Distrito Federal, Aragua, and Carabobo states, among others, and it is estimated that at least 120 people died, several and more than 4,000 victims as a result of this tropical storm [25]. Other catastrophes such as the one that occurred in Vargas state in 1999 and 2005, which destroyed housing and sanitation infrastructure, including the collapse of sewage and water systems, mainly in Vargas, Miranda, and Falcón states [9]. Similarly, in 2005, in the Mocotíes tragedy in Mérida state, land communications were interrupted, among other natural disasters caused by rain in Aragua and Miranda states, with deaths and great destruction of populations such as Araira, with many refugees [10]. The vaccine has had a significant effect in reducing HAV infections, although coverage rates remain lower compared to other childhood vaccines [26]. Countries in the region, such as Argentina, have had successful experiences following the incorporation of the vaccine into the national immunisation schedule, with rates declining sharply from 113.3/100,000 in 2004 to 1.4/100,000 in 2011. Throughout this period, vaccination coverage was over 90% nationwide [27]. In contrast, in Venezuela, in the case of hepatitis A vaccination, it is not included in the Venezuelan Expanded Programme on Immunisation, it is only applied in the private sub-sector and in the years of better economic boom, prior to 2013, it reached between 10% and 15% of vaccination coverage, according to personal and press reports. However, there is no reliable data regarding private sector indicators on vaccination [28]. Finally, the WHO Global Health Observatory [29] shows the most relevant indicators for monitoring VH, such as VHB and VHC, from 2000 to 2020. However, it does not keep records of VHA, morbidity data or VH seroprevalence, which further limits understanding of what is happening in Venezuela.

### VHB mortality and chronic sequelae of VH

The spectrum of acute HBV infection ranges from asymptomatic infection to self-limited hepatitis, to fulminant hepatitis. A third of acute infections in adults are symptomatic, and fulminant hepatitis occurs in less than 1% of cases, with a mortality of about 70% [30]. Chronic HBV infection occurs in approximately 5% of those infected in adulthood, compared to 90% of those infected in early childhood [31, 32]. Therefore, the global incidence of chronic HBV infection is largely due to vertical, mother-to-child, and early childhood transmission, and this is the focus of the WHO elimination plan. This study found a negative correlation between VHB and VH mortality and chronic sequelae of VH; that is, as VHB deaths decrease, deaths from sequelae increase; this correlation may be explained by the way deaths from VHB were reported before 2004, which were probably deaths associated with sequelae of VH. The disruptive drop in VHB deaths and the rise in deaths from sequelae of VH evidenced in 2005 may be explained by the change in coding that begins to consider the basic cause of death. This is supported by the analysis of the mortality yearbooks, where the majority of deaths (90%) by malignant tumour of the liver and intrahepatic bile ducts, non-alcoholic liver fibrosis, and cirrhosis occur in adults older than 44 years, coinciding with the most frequent age group (older than 35 years) where the majority of deaths due to sequelae of VH are found (>96%), suggesting that they are a result of chronic hepatitis.

In 2000, the vaccination schedule against hepatitis B in children under one year of age was intensified, and, in 2001, given the high incidence of HBV infection, a vaccination strategy was launched in new-borns to prevent vertical transmission of this disease. Over the years, coverage has progressively increased, reaching 88% in 2006, which may explain the decrease in both morbidity and mortality due to HBV in the last years of this study period [23]. Therefore, the primary intervention to HBV is the vaccination. However, vaccination coverage rates have been declining in recent years [11], including hepatitis B vaccination, reaching a coverage of 56% in 2021 [23]. Although there was a sudden decrease in VHB mortality and an increase in sequelae of VH since the time of the coding change, we consider that the maintenance of this trend may also be due to the impact of vaccination, given the decrease in deaths in children in correlation with the increase in vaccination coverage. Moreover, we argue that deaths due to sequelae of VH will continue to increase at the expense of the population that did not have access to HBV vaccination and who are currently chronically infected.

### Morbidity and mortality of VHC

Conversely, the majority of cases of acute HCV infection occurs without clinically overt disease, but leads to a chronic infection in around 75–85% of people; over the course of 20–30 years, a proportion of patients will progress to liver cirrhosis and hepatocellular carcinoma [33]. The prevalence of HCV infection varies according to geographical area and different risk groups [34]. In Venezuela, there are few studies aimed at determining the frequency of HCV infection in the general population. However, prevalence of HCV infection has been reported from 0.6% to 2.1% [34, 35]. Most seroprevalence studies in the country have focused mainly on small regions (mainly the city of Maracaibo in the northwest of the country), Amerindians and high-risk groups such as sex workers, haemodialysis patients, and prisoners, with prevalence ranging from 0% to 71% [14, 36–44], with the expected trend of increasing prevalence of HCV infection exposure as a function of age. Similar to VHB, VHC mortality correlated significantly with death from sequelae, suggesting changes in epidemiological reporting from 2004 onwards. In addition, studies in Latin America and the Caribbean have reported several other risk factors for HCV infection and eventual hepatocellular carcinoma in these high-risk groups [45–47]. HCV was the aetiology in one third of 116 Venezuelan patients diagnosed with hepatocarcinoma [48]. However, information on HCV-related sequelae in Venezuela remains very limited.

### Limitations

The limitations of this study are several: (1) the unavailability of bulletin publications after 2016 prevent in country morbidity and mortality analysis from being more up-to-date; (2) data quality limits the certainty of our results, although the bulletins are guided by the ICD-10, reporting is done by professionals from all over the country, in different socio-economic contexts, which probably limited the molecular confirmation of VH; (3) uncertainty about the size of the populations, the denominators of rates that are extrapolated from a census of several years’ duration and during a political and economic crisis of great dimensions that has generated significant migration of Venezuelans, many of them of productive age, with an important impact on the size of the general population. It is estimated that there has been a slow migration since 2000, which intensified in the last 10 years [49]. Finally, the information available on VHD and VHE in the bulletins is null and void, which is why they could not be analysed in this time series. Despite these limitations, the trends in morbidity and mortality behaviour provided by the Venezuela’s Ministry of Health offer a better understanding of the behaviour of VH in the country to guide effective responses to this important public health problem.

## Conclusions

VH is a major burden of morbidity and mortality in Venezuela with an endemic-epidemic trend and an intermediate prevalence for VHA, VHB, and VHC. Our analysis shows: (1) a strong correlation between UVH and VHA cases, suggesting that VHA cases have been classified as UVH; (2) a clear relationship between flood and landslide events in Venezuela and VHA epidemic peaks; (3) a strong negative correlation between VHB deaths and sequelae of VH during the study period, after a change in the reporting of VH deaths from 2004–2005 onwards. Epidemiological information is not published in a timely manner and diagnostic tests are insufficient in primary health services. There is an urgent need to resume epidemiological surveillance of VH and to optimise the classification system for a better understanding of UVH cases and deaths due to sequelae of VHB and VHC.

## Data Availability

All data generated or analysed during this study are included within this article.

## Abbreviations

VH: viral hepatitis
HAV: hepatitis A virus
HBV: hepatitis B virus
HCV: hepatitis C virus
HDV: hepatitis D virus
HEV: hepatitis E virus
WHO: World Health Organisation
HBsAg: hepatitis B surface antigen
anti-HBc: hepatitis B core antibody
ICD-10: International Statistical Classification of Diseases and Related Health Problems, Tenth Revision
TVH: total viral hepatitis
UVH: unspecific viral hepatitis
